# Mechanical and biological evaluation of two fresh pepper varieties

**DOI:** 10.3389/fpls.2025.1542262

**Published:** 2025-04-02

**Authors:** Shanwen Zhang, Shaowen Li, Min Dai, Enhui Lu, Sixing Liu, Linquan Ge, Yongji Zhang, Chunsong Guan, Binxing Xv, Wei Su, Hong Miao

**Affiliations:** ^1^ College of Mechanical Engineering, Yangzhou University, Yangzhou, China; ^2^ Vegetable (root vegetable) Fully Mechanized Research Base, Ministry of Agriculture and Rural Affairs, Yangzhou, China; ^3^ College of Plant Protection, Yangzhou University, Yangzhou, China; ^4^ Cash Crops Research Department, Lixiahe Agricultural Research Institute of Jiangsu Province, Yangzhou, China; ^5^ Nanjing Institute of Agricultural Mechanization, Ministry of Agriculture and Rural Affairs, Nanjing, China; ^6^ Agricultural Technology Integrated Service Center, Yangzhou Bureau of Agriculture and Rural Affairs, Yangzhou, China

**Keywords:** pepper biological parameters, pepper mechanical properties, fresh pepper, mechanical harvesting, mechanization-adapted variety

## Abstract

The operation of roller-type pepper harvesters involves striking and pulling the peppers, which may result in incomplete detachment from their stems or cause surface damage or breakage of the peppers. The quality of the harvested peppers is directly influenced by the forces applied during striking and pulling. Therefore, the physical and mechanical properties of peppers are crucial for determining the structural and dynamic parameters of the screw rollers. This study selected Round peppers and the 'Bo 15' line pepper as experimental subjects. Growth parameters such as main stem diameter and fruit diameter were measured. And mechanical properties including tensile, bending, compressive, and shear strength were tested. Results showed that: two pepper varieties had a moisture content of 90% ± 1%. The main stem diameters of Round pepper and 'Bo 15' line pepper were 10.196 ± 1.508 mm and 13.44 ± 0.769 mm. The average diameter of round pepper was greater than that of 'Bo 15' line pepper. For mechanical stress, the 'Bo 15' line pepper exhibited stronger resistance, and the mechanical properties were as follows: tensile strength was 0.83 MPa, bending strength was 0.58 MPa, radial compressive strength was 0.25 MPa, and shear strength was 0.28 MPa. This study provides a basis for the design of low damage harvesting device for fresh peppers and the selection of varieties suitable for mechanical harvesting.

## 1 Introduction

China is the world’s largest producer and consumer of chili peppers, with a cultivation area of approximately 814,000 hectares and an annual production of 19.6 million tons, ranking among the highest globally (Zhang, 2023). There are three main types of pepper products: fresh peppers, dried peppers, and processed peppers. Fresh peppers are primarily cultivated in regions such as Shandong, Hebei, and Inner Mongolia, while dried peppers are mainly distributed in Yunnan, Guizhou, Sichuan, and Xinjiang. Processed peppers are primarily grown in Gansu and Shaanxi (Wang et al., 2010, Wang et al., 2021). With the increasing demand for pepper production, the quality requirements for harvesting are also rising. The harvesting machines for dried peppers are mainly drum-type, which have reached a mature level of mechanization. In contrast, fresh pepper harvesters are primarily roller-type, but issues such as high harvesting damage rates and high impurity levels still persist. Therefore, improving the mechanization quality of fresh pepper harvesting is crucial (Jin et al., 2023).

The operation of roller-type pepper harvesters primarily involves striking and pulling the peppers using roller components. However, this process can cause pepper fruits to undergo compression and bending. If the force exerted by the rollers is great, it can lead to damage or breakage of the pepper fruits. Conversely, if the force is weak, the pepper fruits will not detach from the stems, making harvesting impossible. Therefore, selecting the appropriate roller force is crucial. The force that pepper fruits can withstand plays a decisive role in determining the roller force, highlighting the need to study the mechanical properties of pepper fruits (Akinoso et al., 2014; Yuan et al., 2021; Jia et al., 2023; Almady et al., 2024; Chen et al., 2024). Currently, research on the mechanical properties of peppers primarily focuses on dried peppers. For example (Zhang et al., 2024), conducted experimental measurements on the physical and mechanical properties of millet pepper, reporting a tensile strength of 0.68 MPa and a shear strength of 0.32 MPa. These findings provide valuable data references for the mechanized harvesting of millet pepper. With the assistance of shear and tensile mechanical tests (Li et al., 2024), determined the shear and tensile strengths of the pedicle of flat pepper to be 9.73 MPa and 5.13 MPa, respectively. These results lay a foundation for the development of mechanized pedicle removal equipment for peppers. This study draws on research methodologies applied to millet pepper and flat pepper. Two varieties of fresh peppers—Round pepper and the ‘Bo 15’ line pepper—were selected for physical measurements and mechanical testing. Round pepper and the ‘Bo 15’ line pepper represent two extreme morphological types: Round peppers exhibit a short, barrel-like shape, while the ‘Bo 15’ line pepper displays a slender, conical form. This morphological difference makes them ideal counterparts for studying the influence of geometric morphology on mechanical properties, providing a basis for low damage harvesting of fresh peppers and the selection of varieties suitable for mechanical harvesting.

## 2 Materials and methods

### 2.1 Materials

**Figure 1:**
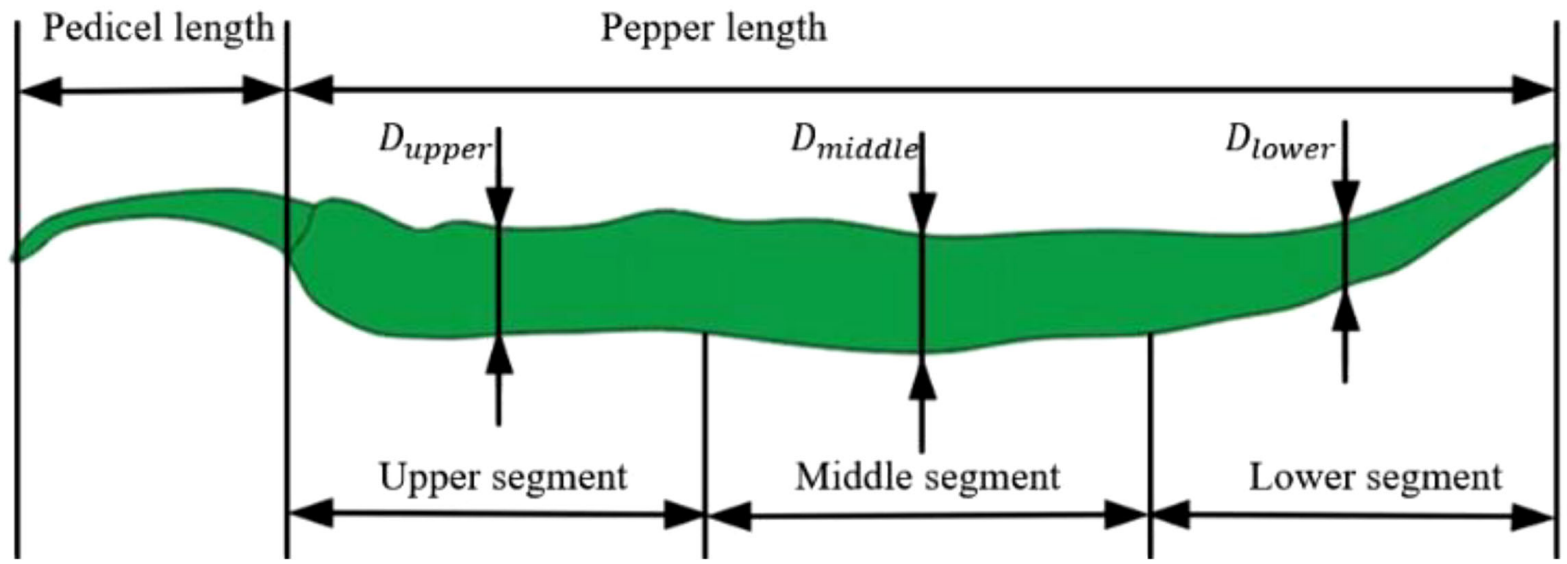
Pepper structure.

The fresh pepper samples used in this experiment included Round peppers and the ‘Bo 15’ line pepper. The experimental samples were provided by the Modern Agricultural Industrial Park in Hanjiang District, Yangzhou City. All peppers were cultivated in greenhouses with strictly regulated temperature (25 ± 2°C), humidity (70 ± 5%), and irrigation schedules (uniform drip irrigation), and were planted in March 2024. After the plants of the two pepper varieties were harvested from the experimental field, the experiments were conducted immediately to determine their mechanical properties, ensuring that the experiments were completed within 24 hours. After 24 hours, resampling was performed for additional experiments. As shown in **Figure 1**, each pepper fruit was cut at the pedicel end and divided into three sections: the upper segment, middle segment, and lower segment, from left to right. Due to the irregular shape of the pepper fruits, the diameter at the middle position of each segment was measured and recorded as the diameter of that segment.

### 2.2 Sample preparation

#### 2.2.1 Biological parameters and moisture content of pepper fruits

**Figure 2:**
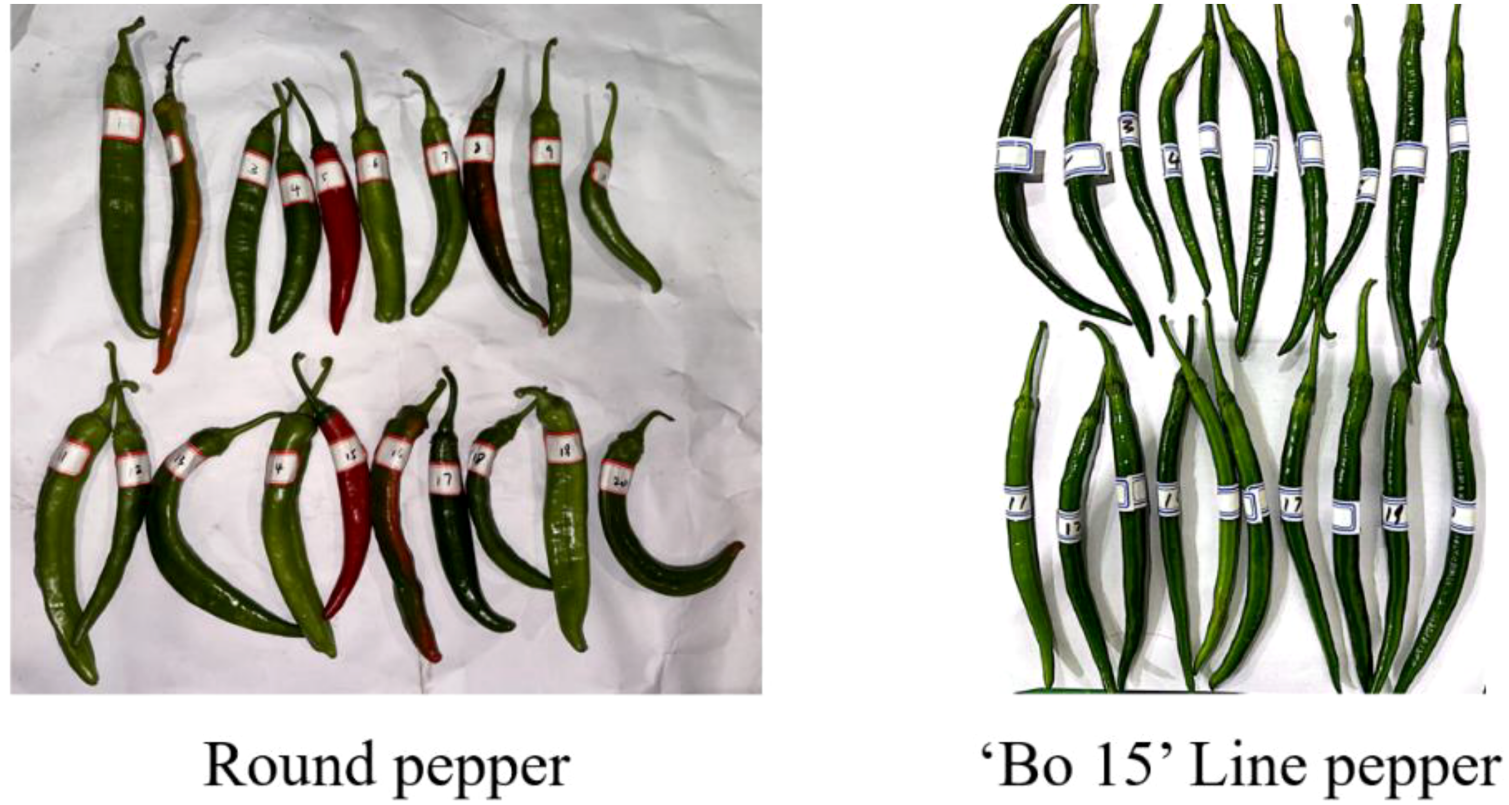
Measurement of biological parameters of pepper fruit samples.

**Figure 2** shows that 20 pepper samples were randomly selected from each variety. The physical properties of the two pepper varieties were measured using the following instruments: a measuring tape (accuracy: 1 mm), an electronic balance (accuracy: 0.0001 g), and a vernier caliper (accuracy: 0.02 mm). For each variety, 20 sets of data were collected, and the average values of these measurements were used for subsequent analysis. This ensures a reliable assessment of the physical characteristics of each pepper variety.

According to the method of drying with an oven (O’Kelly, 2004), segments of 20 mm in length were cut from the upper, middle, and lower segments of the pepper fruits and weighed using an electronic balance. The initial weight was recorded as m1. The pepper segments were then placed in a petri dish and dried in a forced-air drying oven at 65°C for 10 hours, followed by a weighing process. After the initial drying, the samples were returned to the drying oven for an additional two hours and then reweighed. This cycle was repeated until the difference between two consecutive weight measurements was less than 0.005 g, indicating that drying was complete. The dried pepper segments were then removed from the oven and allowed to return to room temperature before a final weight measurement was taken, recorded as m2. The moisture content of the pepper was calculated using the following Equation (1) (Moya-Ignacio et al., 2024).


(1) $$\begin{array}{c} W=\frac{m_{1}-m_{2}}{m_{1}}\times 100\% \end{array}$$

Where W is the moisture content (%), *m*_1_ is the mass before drying (g), and *m*_2_ is the mass after drying (g).

#### 2.2.2 Samples for mechanical property testing of pepper fruits

Fifteen peppers with intact pedicels were randomly selected from each variety to measure the connection force between the pedicel and the fruit.

Additionally, 15 peppers with the pedicel removed were randomly selected from each variety for tensile mechanical performance testing. Another 15 peppers with the pedicel removed were selected for bending mechanical performance testing, with the results used to calculate the bending strength and bending elastic modulus (Butiter et al., 2013; Wang and Zhang, 2015).

For radial compression mechanical performance testing, 15 complete pepper fruits were randomly selected from each variety, and 20 mm segments from the upper, middle, and lower parts of the fruits were used as test samples (Song et al., 2022).

**Figure 3:**
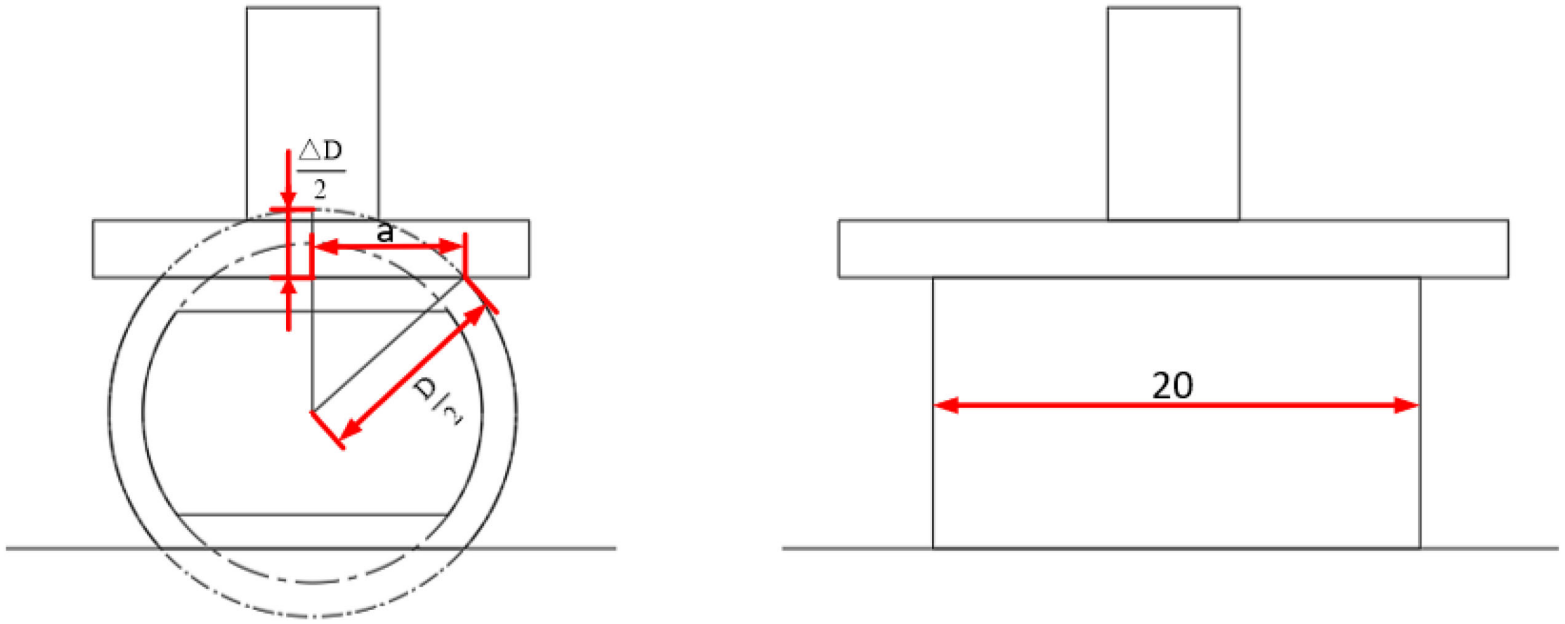
Schematic diagram of the radial compression test.


**Figure 3** shows that the contact surface for radial compression of the pepper can be considered a rectangle with a length of 20 mm and a width of 2a, as shown in Equation (2) below:

(2) $$\begin{array}{c} 2a=2\sqrt{{\left(\frac{\text{D}}{2}\right)}^{2}-{\left(\frac{\text{D}-\text{ΔD}}{2}\right)}^{2}}=\sqrt{2\text{D}\cdot \text{ΔD}-{\text{ΔD}}^{2}} \end{array}$$

Where D is the diameter of the pepper (mm) and ΔD is the deformation of the pepper (mm).

The area of the radial compression contact surface is calculated as shown in Equation (3) below:


(3) $$\begin{array}{c} S=\sqrt{2\text{D}\cdot \text{ΔD}-{\text{ΔD}}^{2}}\cdot 20 \end{array}$$

Where S is the area of the radial compression contact surface (mm^2^).

Using the radial compressive strength formula, the radial compressive strength of the pepper fruit was calculated, and the elastic modulus for different parts of the pepper was obtained (Wang et al., 2024).

Additionally, 15 complete pepper fruits were randomly selected from each variety, and 40 mm segments were cut from the upper, middle, and lower parts to serve as samples for shear mechanical performance testing. The shear strength of the peppers was calculated based on the measured shear force (Jahanbakhshi et al., 2020).

#### 2.2.3 Samples for measuring growth parameters of pepper plants

Five pepper plants from each variety, free of pests, diseases, and visible damage, were randomly selected as experimental samples. The height of the pepper plants and the fruiting status can significantly impact mechanized harvesting (Alibas and Koksal, 2015). If the heights of the pepper plants are uniform (Wang et al., 2023a), it eliminates the need to adjust the height of the harvesting machine’s working components, greatly improving the efficiency of mechanized harvesting. Additionally, the lower the fruiting position of the peppers, the faster they mature and the higher the yield; however, harvesting at lower positions can increase the risk of impurities being mixed in, thus increasing the workload for post-harvest sorting (Peng et al., 2023; Wang et al., 2023b).

#### 2.2.4 Samples for testing the connection force between peppers and stems

Five healthy pepper plants without pests, diseases, or visible damage were randomly selected from each variety as experimental samples. To avoid the impact of prolonged storage on the experimental data, the connection force measurement must be conducted within 24 hours of harvesting the pepper plants to ensure the accuracy of the experiment.

### 2.3 Experimental methods

The experiments were conducted using a DK-10KN universal testing machine produced by Deka Precision Meter Co., Ltd, with the stress-displacement curves read through the corresponding control software. This testing machine can apply forces of varying magnitudes and directions, as well as different testing speeds; calibration must be performed before each experiment. The measured results were imported into SPSS, and a one-way ANOVA (analysis of variance) was performed to analyze the mechanical properties of the two pepper varieties.

#### 2.3.1 Connection force test between pedicel and fruit

The pedicel and fruit tip of the pepper samples were clamped using the tensile fixture of the DK-10KN universal testing machine (Ortega et al., 2024), with a tensile speed set at 10 mm· min^-^¹. To prevent the pepper from slipping out of the fixture during measurement, medical tape was used to wrap the pedicel and the fruit tip (Cao et al., 2023). The tensile force increases as the displacement increases, and when the pedicel separates from the pepper fruit, the tensile force drops sharply, marking the end of the experiment.

#### 2.3.2 Tensile force test

The upper and lower ends of the pepper fruit samples were clamped using the tensile fixture of the DK-10KN universal testing machine, with a tensile speed set at 10 mm·min^-^¹. To prevent the pepper fruit samples from slipping out of the fixture during measurement, medical tape was used to wrap the upper and lower ends of the samples. At the start of the experiment, the tensile force increases with the displacement, reaching its maximum when the pepper fruit fractures, marking the end of the experiment (Yv et al., 2021).

#### 2.3.3 Bending force test

A three-point bending method was used to conduct bending experiments on 15 randomly selected whole fresh peppers of each variety (Yang et al., 2020). During the experiment, the peppers were placed horizontally on the supports of the bending fixture equipped with the measuring instrument, with a distance of 80 mm between the two supports. The bending knife’s movement speed was set at 10 mm· min^-^¹ (Ma et al., 2022). At the start of the experiment, the pressure increases with the dis-placement, reaching its maximum pressure when the pepper fruit fractures, marking the end of the experiment.

#### 2.3.4 Radial compression force test

During the experiment, the pepper segments were placed horizontally on the compression plate aligned with the axis, and the moving speed of the compression plate was set to 10 mm·min^-^¹ (Wang and Gao, 2018). At the be-ginning of the experiment, the pressure increased with the displacement, reaching the maximum pressure value when the pepper segment sample fractured (Wei et al., 2021).

#### 2.3.5 Shear force test

During the experiment, the pepper segments were placed horizontally in the shear fixture of the instrument and fixed in place with medical tape (Desmet et al., 2002). The moving speed of the shear blade is set to 10 mm·min^-^¹. At the beginning of the experiment, the pressure increases with the displacement of the blade, and the maximum pressure is reached when the surface of the pepper segment is cut through by the blade, marking the end of the experiment (Zhao et al., 2021).

#### 2.3.6 Connection force test of pepper and stem

The connection force of the pepper was measured using a Handpi tensile and compressive testing machine. During the experiment, the tail end of the pepper was wrapped with medical tape, and the connection force between the pepper and the stem was measured at angles of 0°, 45°, 90°, 135°, and 180°. 0° indicates that the force direction is aligned with the pepper’s growth direction, while 180° indicates that the force direction is opposite to the pepper’s growth direction.

## 3 Results

### 3.1 Results of biological parameters and moisture content of peppers

The biological parameters and average moisture content of each pepper variety are shown in **Table 1**. After harvesting, the pepper fruits must be promptly wrapped and stored to prevent moisture loss, which could affect the quality of the peppers.

**Table 1:** Biological parameters and moisture content of pepper fruits.

Variety	Length (mm)	Pedicel Length (mm)	Mass (g)	Section	Diameter (mm)	Moisture Content
Round Pepper	184.94	51.28	34.4295	Upper	21.92	90.24%
				Middle	19.50	90.57%
				Lower	15.06	90.26%
‘Bo 15’ Line Pepper	171.94	43.30	12.1672	Upper	10.40	90.85%
				Middle	12.24	89.81%
				Lower	6.030	90.13%

### 3.2 Results of the fruit pedicel and fruit connection force test

**Table 2** presents the results of the connection force test between the pepper pedicel and fruit. The results indicate that there is no significant difference in the pedicel-fruit connection strength between Round peppers and ‘Bo 15’ line peppers.

**Table 2:** Results of the connection force test between pepper pedicel and fruit.

Variety	Pedicel-Fruit Connection Force (N)
Round Pepper	35.86 ± 1.718
‘Bo 15’ Line Pepper	34.96 ± 1.815
F-Value	1.957
p-Value	0.173

### 3.3 Tensile strength test results

According to the tensile stress measurement results of peppers in **Table 3**, the tensile strength of the two types of peppers showed significant differences, the tensile strength of the two types of peppers is as follows: ‘Bo15’ line pepper > Round pepper. Therefore, during mechanical harvesting, ‘B15’ line pepper are less likely to be damaged. Additionally, the tensile stress applied during pepper harvesting should be kept below the minimum stress to avoid fracture and ensure the quality of the harvest.

**Table 3:** Tensile stress measurement results of peppers.

Variety	Breaking Force (N)	Cross-sectional Area (mm²)	Tensile Strength (MPa)
Round Pepper	86.00	131.58	0.6536 ± 0.048^b^
‘Bo 15’ Line Pepper	36.02	43.16	0.8346 ± 0.1029^a^
F-Value			38.072
p-Value			<0.001

### 3.4 Bending force test results

The bending strength of the fruits of the two types of peppers showed significant differences. **Table 4** shows that the relationship of elastic modulus for the two types of peppers is: ‘Bo 15’ line pepper > Round pepper. The order of bending strength is also: ‘Bo 15’ line pepper > Round pepper. Therefore, ‘Bo 15’ line pepper is less likely to be bent or broken during mechanized harvesting.

**Table 4:** Measurement results of bending stress of peppers.

Variety	Bending Stress (N)	Deflection (mm)	Elastic Modulus (MPa)	Bending Strength (MPa)
Round Pepper	19.06	21.73	0.2592 ± 0.0303^b^	0.2416 ± .00412^b^
‘Bo 15’ Line Pepper	5.82	20.09	0.5174 ± 0.2442^a^	0.5821 ± 0.1568^a^
F-Value			16.522	66.195
p-Value			<0.001	<0.001

### 3.5 Results of radial compression force test

As shown in **Table 5**, there is no significant difference in the radial compressive strength between the middle and lower segments of the Round pepper fruit. However, the upper segment of the Round pepper shows a significant difference compared to the other two segments. In the ‘Bo 15’ line pepper fruit, no significant differences in radial compressive strength were observed among the upper, middle, and lower segments.

**Table 5:** The radial compression mechanical properties of the two pepper fruit varieties.

Variety	Mechanical Parameter	Upper Segment	Middle Segment	Lower Segment	F-Value	P-Value
Round Pepper	Radial Compression Load (N)	43.14	33.26	35.56		
	Radial Compression Elastic Modulus (MPa)	0.4047 ± 0.0547^a^	0.3248 ± 0.0256^c^	0.3588 ± 0.0344^b^	15.007	<0.001
	Radial Compression Strength (MPa)	0.1355 ± 0.0244^a^	0.1083 ± 0.0125^b^	0.1211 ± 0.0156^b^	8.363	<0.001
‘Bo 15’ Line Pepper	Radial Compression Load (N)	45.28	48.48	56.53		
	Radial Compression Elastic Modulus (MPa)	1.0244 ± 0.3186	1.1306 ± 0.5087	0.9295 ± 0.3365	0.962	0.391
	Radial Compression Strength (MPa)	0.2506 ± 0.0734	0.2603 ± 0.0654	0.2412 ± 0.0580	0.315	0.732

As can be clearly seen in **Table 6**, there are significant differences in the radial compressive strength among the two types of peppers. The radial compressive strength of the ‘Bo 15’ line pepper fruit is generally greater than that of the Round pepper. Therefore, the ‘Bo 15’ line pepper fruit is more resistant to crushing under radial compressive force.

**Table 6:** Presents the analysis of the compressive strength of the two types of peppers.

Variety	Sample size	Radial Compression Strength (MPa)	Radial Compression Elastic Modulus (MPa)
Round Pepper	45	0.1216 ± 0.0210^b^	0.3628 ± 0.0513^b^
‘Bo 15’ Line Pepper	45	0.2507 ± 0.0648^a^	1.0281 ± 0.3970^a^
F-Value		161.505	124.336
p-Value		<0.001	<0.001

Lowercase letters in the table indicate significant differences between groups.

### 3.6 Shear force test results

As shown in **Table 7**, there are significant differences in shear strength among the upper, middle, and lower segments of the Round pepper fruit, with the shear strength ordered as follows: lower segment > upper segment > middle segment. In the ‘Bo 15’ line pepper fruit, no significant difference in shear strength was observed between the upper and lower segments, but the middle segment showed a significant difference from the other two segments. **Table 8** shows that there are significant differences in shear strength among the two types of peppers, the shear strength of ‘Bo 15’ line pepper is generally higher than that of Round pepper, making it more resistant to shear force.

**Table 7:** The shear mechanical properties of the two pepper fruit varieties.

Variety	Mechanical Parameter	Upper Segment	Middle Segment	Lower Segment	F-Value	P-Value
Round Pepper	Shear Force (N)	38.97	29.84	30.38		
	Shear Strength (MPa)	0.0995 ± 0.0153^b^	0.0851 ± 0.0125^c^	0.1136 ± 0.025^a^	9.044	<0.001
‘Bo 15’ Line Pepper	Shear Force (N)	19.57	16.24	8.23		
	Shear Strength (MPa)	0.3231 ± 0.0585^a^	0.2294 ± 0.0413^b^	0.2940 ± 0.0556^a^	12.586	<0.001

Lowercase letters in the table indicate significant differences between groups.

**Table 8:** presents the analysis of shear strength for the two types of peppers.

Variety	Sample size	Shear Strength (MPa)
Round Pepper	45	0.0994 ± 0.0215^b^
‘Bo 15’ Line Pepper	45	0.2822 ± 0.0647^a^
F-Value		323.542
p-Value		<0.001

Lowercase letters in the table indicate significant differences between groups.

### 3.7 Results of pepper plant growth parameter measurement

The growth parameters of pepper plants primarily include plant width, stem diameter, height, and fruiting position. Five plants each of the Round pepper and ‘Bo 15’ line pepper varieties were randomly selected for measurement, as shown in **Table 9**. Data revealed significant differences in main stem diameter and fruiting height between the two varieties. The main stem of “Bo 15” was notably thicker than that of Round pepper, and both the minimum and maximum fruiting heights of Round pepper plants were lower than those of ‘Bo 15’ line pepper. Specifically, the average main stem diameter was 10.196 ± 1.508 mm for Round pepper and 13.44 ± 0.769 mm for ‘Bo 15’ line pepper. The minimum fruiting height for Round pepper was 355.4 ± 47.443 mm, with a maximum of 676.2 ± 84.591 mm, while for ‘Bo 15’ line pepper, these values were 429 ± 46.266 mm and 831.2 ± 87.947 mm, respectively. No significant differences were observed in plant width, height, or fruit number between the two varieties. These findings provide a preliminary reference for designing the operational components of a pepper harvesting machine.

**Table 9:** The growth parameters of the two pepper plant varieties.

Variety	Main Stem Diameter (mm)	Maximum Width (mm)	Maximum Height (mm)	Fruit Count	Effective Branch Count	Minimum fruiting height(mm)	Maximum fruiting height (mm)
Round Pepper	10.196 ± 1.508	840.6 ± 85.772	947.2 ± 45.141	23 ± 4.301	13.8 ± 1.643	355.4 ± 47.443	676.2 ± 84.591
‘Bo 15’ Line Pepper	13.44 ± 0.769	792.0 ± 46.632	982.2 ± 83.209	30 ± 6.708	16.2 ± 4.087	429 ± 46.266	831.2 ± 87.947
F-Value	18.357	1.239	0.683	3.858	1.485	6.168	8.067
p-Value	0.003	0.298	0.432	0.085	0.258	0.038	0.022

### 3.8 Results of the connection strength test between pepper pedicels and stems

**Table 10** shows that the connection strength between the pedicels and stems of Round pepper is maximal at 90° and decreases as the angle shifts to either side, while the connection strength between the pedicels and stems of ‘Bo 15’ line pepper shows no significant correlation with the angle. The average connection strength for Round pepper is 11.79 N, whereas for ‘Bo 15’ line pepper it is 8.04 N. This indicates that the pedicels of ‘Bo 15’ line pepper are easier to harvest compared to those of Round pepper. Additionally, from the previous experiments on the connection strength between the pedicels and fruits, it can be noted that both types have lower connection strengths with the stems than with the fruits.

**Table 10:** The connection strength between the pedicels and stems of the two pepper varieties.

Variety	0°	45°	90°	135°	180°	F-Value	P-Value
Round Pepper	9.09 ± 0.721^d^	12.864 ± 1.521^b^	17.414 ± 1.33^a^	12.006 ± 2.023^b^	9.592 ± 1.969^c^	29.707	<0.001
‘Bo 15’ Line Pepper	4.518 ± 0.358^b^	8.756 ± 2.266^a^	8.546 ± 0.797^a^	8.152 ± 2.932^a^	10.234 ± 2.776^a^	5.067	0.006

Lowercase letters in the table indicate significant differences between groups.

As the focus of this study is on the mechanical properties of pepper fruits, a limited number of samples were selected for measuring the biological parameters of pepper plants. The findings regarding the biological parameters of pepper plants in this study are preliminary, and more in-depth research with an expanded sample size is required.

## 4 Conclusions

Two types of fresh peppers—Round pepper and ‘Bo 15’ line pepper—were selected for investigation of their biological parameters and mechanical. The average lengths measured were 184.94 mm for Round pepper and 171.94 mm for ‘Bo 15’ line pepper. The average weights were 34.43 g and 12.17 g, respectively. Mechanical property parameters were obtained through tensile, bending, compression, and shear tests. The experimental results indicate that:

1. In the pedicel-fruit connection force test, the average connection force for Round pepper and ‘Bo 15’ line pepper was as follows: Round pepper (35.86 N) > ‘Bo 15’ line pepper (34.96 N).
2. In the tensile test, the average tensile strength of Round pepper and ‘Bo 15’ line pepper was as follows: ‘Bo 15’ line pepper (0.83 MPa) > Round pepper (0.65 MPa).
3. In the bending test, the bending strength of Round pepper and ‘Bo 15’ line pepper was as follows: ‘Bo 15’ line pepper (0.58 MPa) > Round pepper (0.24 MPa). The bending elastic modulus was: ‘Bo 15’ line pepper (0.52 MPa) > Round pepper (0.26 MPa).
4. In the radial compression test, Round pepper exhibited maximum radial compressive strength and elastic modulus in the upper segment, at 0.14 MPa and 0.40 MPa, respectively; ‘Bo 15’ line pepper had maximum radial compressive strength and elastic modulus in the middle segment, at 0.26 MPa and 1.13 MPa.
5. In the shear test, the maximum shear strength of Round pepper occurred in the lower segment, at 0.11 MPa; while ‘Bo 15’ line pepper’s maximum shear strength occurred in the upper segment, at 0.32 MPa.
6. In the connection force test between the pedicel and the stem, the connection force of Round pepper was greatest at 90°, decreasing with angle changes to either side, while the connection force of ‘Bo 15’ line pepper showed no significant relationship with angle. The maximum connection force for Round pepper was 17.41N, and for ‘Bo 15’ line pepper, it was 10.23N.

Overall, under the condition of equal moisture content in both pepper varieties, in the connection force tests between the pedicel and fruit, the connection strength between the pedicel and fruit of Round pepper and ‘Bo 15’ line pepper is similar. In terms of resisting mechanical stress, ‘Bo 15’ line pepper is stronger than Round pepper. In terms of growth parameters and testing the connection strength between the pedicel and stem, the minimum fruit-hanging height for Round pepper is approximately 355 mm, while for ‘Bo 15’ line pepper, it is approximately 429 mm. Both types of peppers have a connection force between the pedicel and stem that is less than that between the pedicel and fruit, making them easy to harvest with the pedicel. The physical parameters and mechanical properties of these two types of pepper will provide a basis for low damage harvesting of fresh peppers and the selection of suitable mechanized varieties.

## 5 Discussion

(Kopta et al., 2020; Chen et al., 2024)demonstrated that moisture content significantly affects the mechanical properties of pepper fruits. In this study, we conducted moisture content tests on both types of peppers, and the results showed that their moisture content was within the range of 90% ± 1%, and all experiments were conducted under this condition to ensure consistency.

(Weryszko-Chmielewska and Michałojć, 2012; Zhigila et al., 2014) noted structural variations among pepper fruits and within different sections of the same fruit. Accordingly, we segmented the fruits into upper, middle, and lower parts to assess compressive and shear strengths. The upper section of Round pepper exhibited a compressive strength of 0.1355 ± 0.0244 MPa, significantly higher than the middle and lower sections (p< 0.05), whereas ‘Bo 15’ line pepper showed uniform compressive strength across all sections at approximately 0.25 MPa (p > 0.05), indicating greater structural stability.

For shear strength, Round pepper displayed significant differences across sections (p< 0.05), peaking at 0.1136 ± 0.025 MPa in the lower section. In contrast, ‘Bo 15’ line pepper showed no significant variation between upper and lower sections (approximately 0.3 MPa, p > 0.05), with the middle section at 0.2294 ± 0.0413 MPa (Rokayya and Khojah, 2016). attributed such mechanical disparities to fruit structural characteristics, these results indicate that the fruit structure of ‘Bo 15’ line pepper is more stable than that of round pepper in both compressive and shear tests.

(Han et al., 2024) stated in his study that the harvesting device of the pepper harvesting machine needs to be designed based on the biological parameters of the pepper plants. We also evaluated biological parameters, including main stem diameter, fruit number, fruiting height, and plant height. The main stem diameter of Round pepper (10.196 ± 1.508 mm) was significantly smaller than that of ‘Bo 15’ line pepper (13.44 ± 0.769 mm, p< 0.05). However, plant height for both varieties was similar, averaging 950 mm (p > 0.05), consistent with (Peng et al., 2023). Fruit number showed no significant difference between varieties (p > 0.05), but ‘Bo 15’ line pepper exhibited a higher fruiting height than Round pepper, aligning with (Wang et al., 2023b). Based on this information, the length of the spiral rollers can be initially designed.

The rotation of roller structures generates tapping or pulling forces, enabling the harvesting of pepper fruits. This relationship can be expressed by Equation (4) below:


(4) $$\begin{array}{c} \text{F}={\text{mw}}^{2}\text{r}=4m\pi^{2}{\text{n}}^{2}\text{r} \end{array}$$

Taking ‘Bo 15’ line pepper as an example, the attachment force between the pedicel and the stem ranges from 4.04 to 12.98 N. This indicates that when the tapping or pulling force falls within this range, the pepper fruit can be harvested. Based on Equation (4), the roller rotation speed can be calculated using Equation (5) below:


(5) $$\begin{array}{c} \text{n}=\sqrt{\frac{\text{F}}{{4m\pi }^{2}\text{r}}} \end{array}$$

Where F is the tapping or pulling force (N), m is the mass of peppers in contact with the roller during harvesting (kg). n is the roller rotation speed (r/min), and r is the radius of the roller (m).

Taking the ‘Bo 15’ line pepper as an example, assuming a spiral roller diameter of 100 mm and a simultaneous harvesting of 10 peppers, the rotation speed of the roller must reach at least 310 r/min according to Formula (5) (Kang et al., 2016) utilized a roller speed of 400 r/min for harvesting fresh Longgreenmat variety peppers, which is similar to the results of this study. To account for potential force measurement errors during the mechanical property tests and ensure reliability and accuracy, the roller rotation speed range may be slightly expanded when the harvester operates.

## Data Availability

The raw data supporting the conclusions of this article will be made available by the authors, without undue reservation.
