# Supplementary material for: Mechanical and biological evaluation of two fresh pepper varieties
Source: Front Plant Sci. 2025 Apr 2;16:1542262. doi: 10.3389/fpls.2025.1542262 (PMC11999842; doi:10.3389/fpls.2025.1542262)
Supplement: Supplementary file 1 [file DataSheet1.docx]

Supplementary Material

## Supplementary Figures


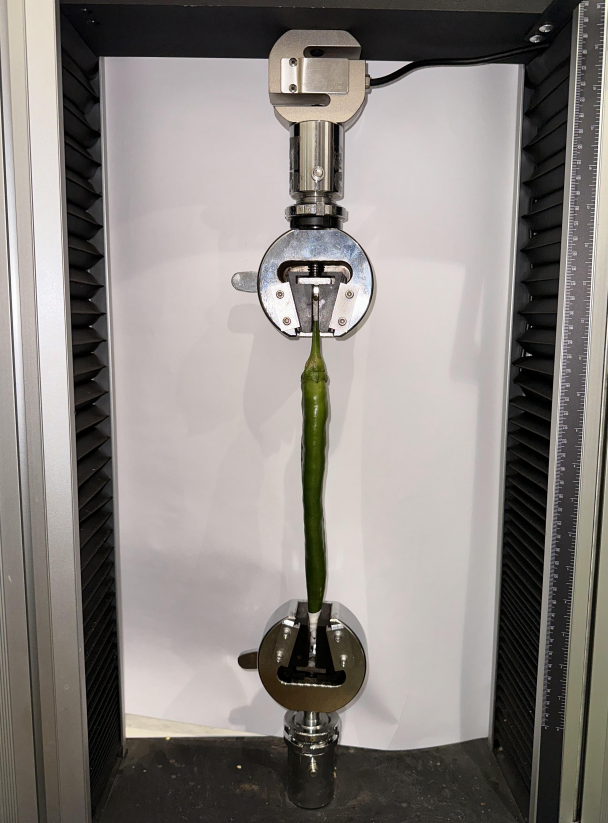


Supplementary Figure 1: Experiment for Measuring the Connection Force Between Pepper Pedicel and Fruit.


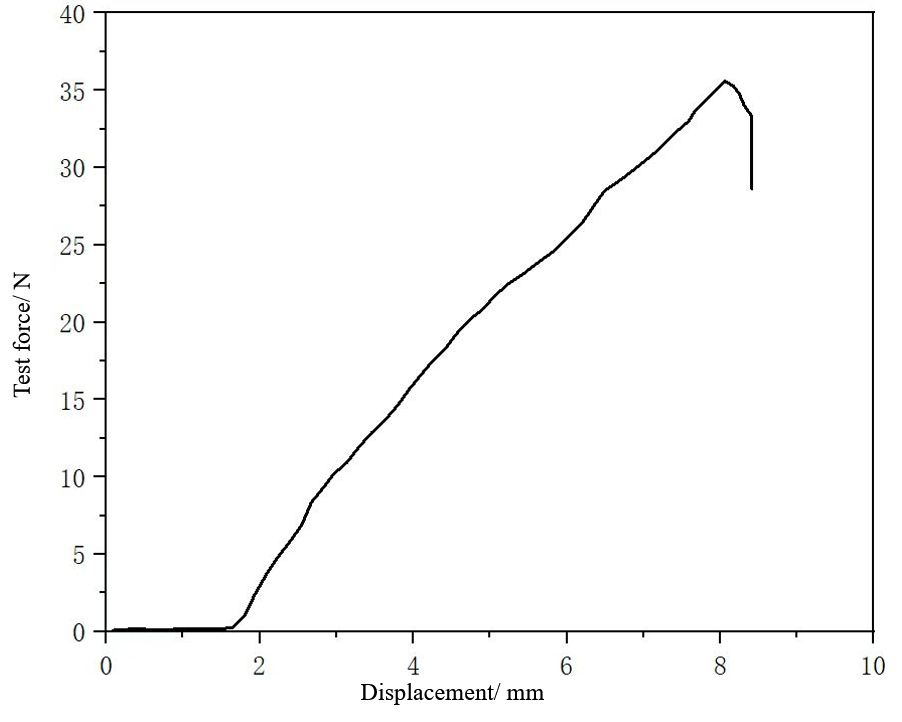


Supplementary Figure 2: Stress-Strain Curve of the Connection Between Pepper Pedicel and Fruit.


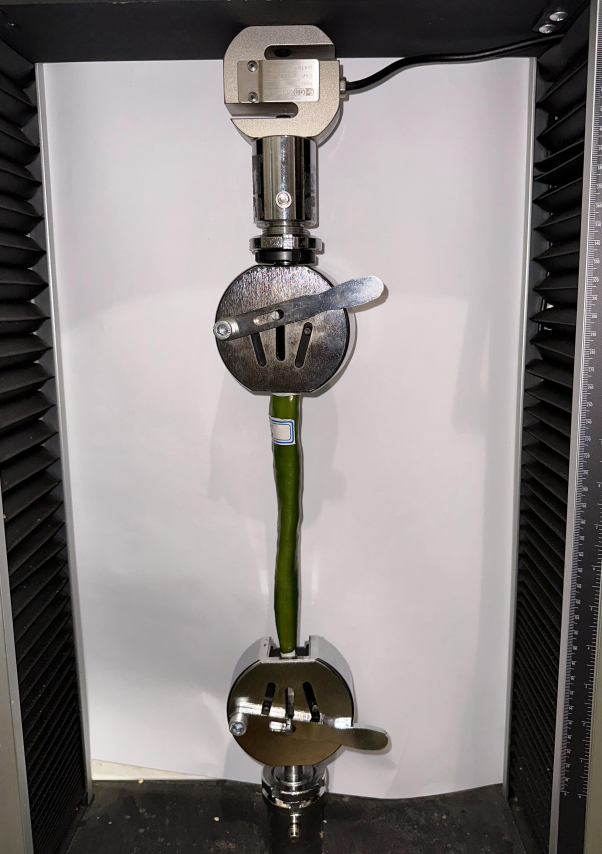


Supplementary Figure 3: Tensile Test of Pepper Fruit.


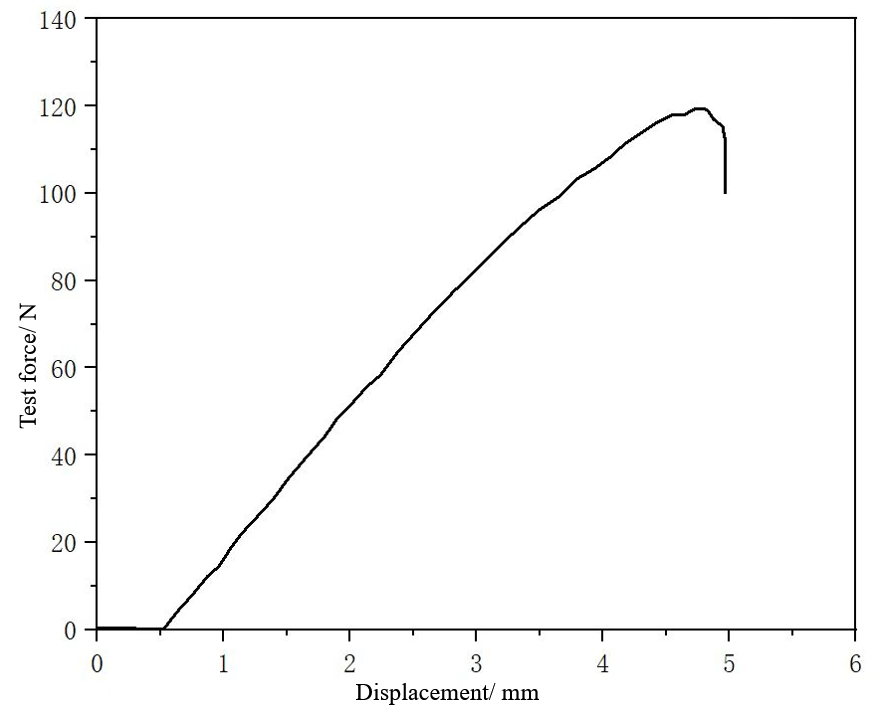


Supplementary Figure 4: Tensile Stress-Strain Curve of Pepper Fruit.


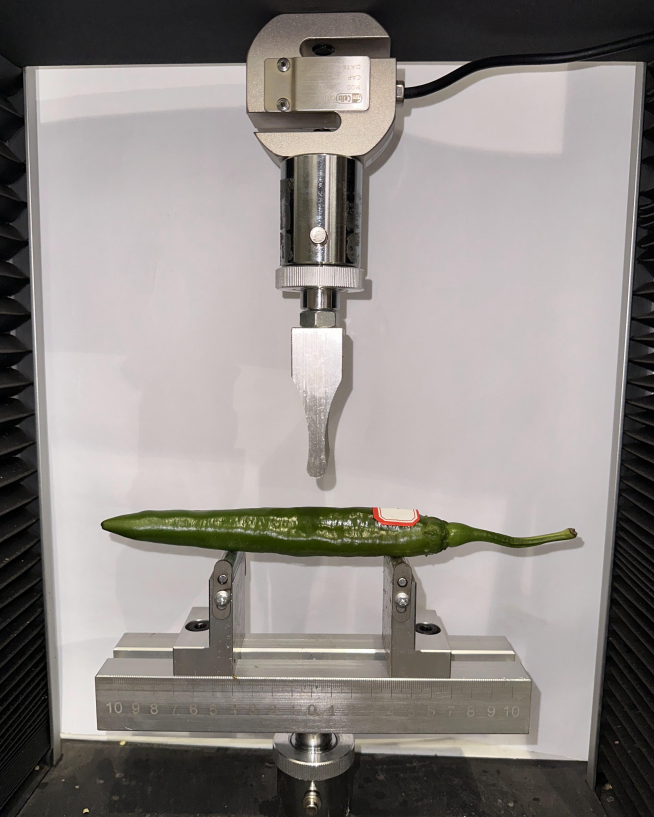


Supplementary Figure 5: Bending Test of Pepper.


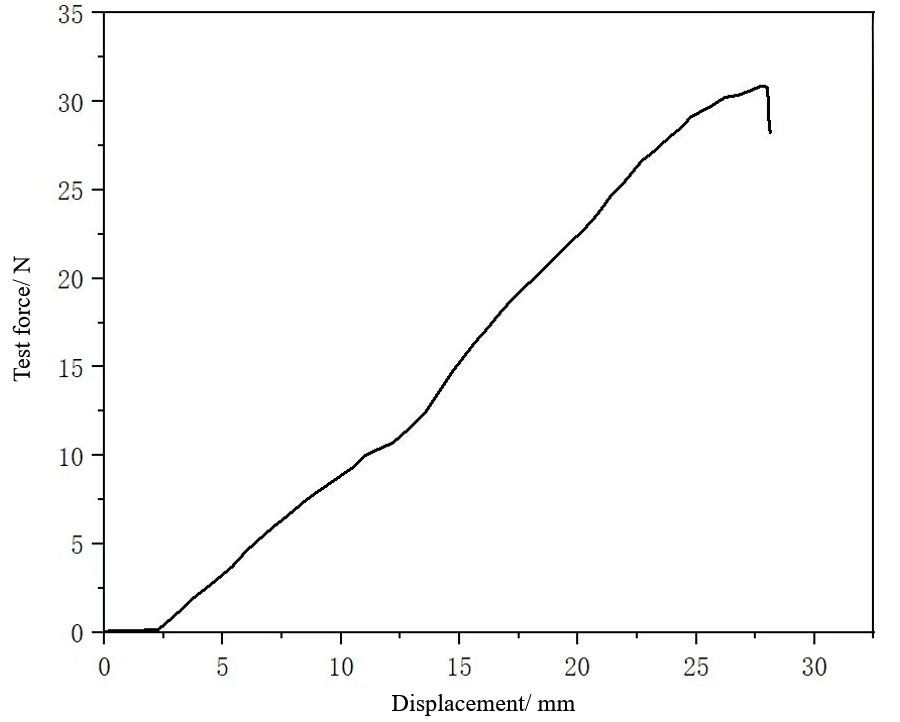


Supplementary Figure 6: Stress-Strain Curve of Bending Test.


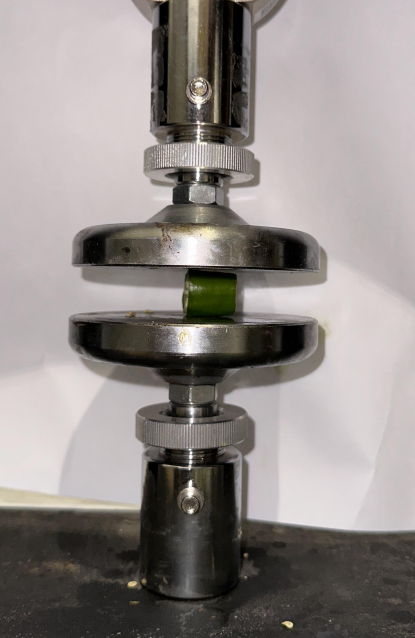


Supplementary Figure 7: Radial Compression Test.


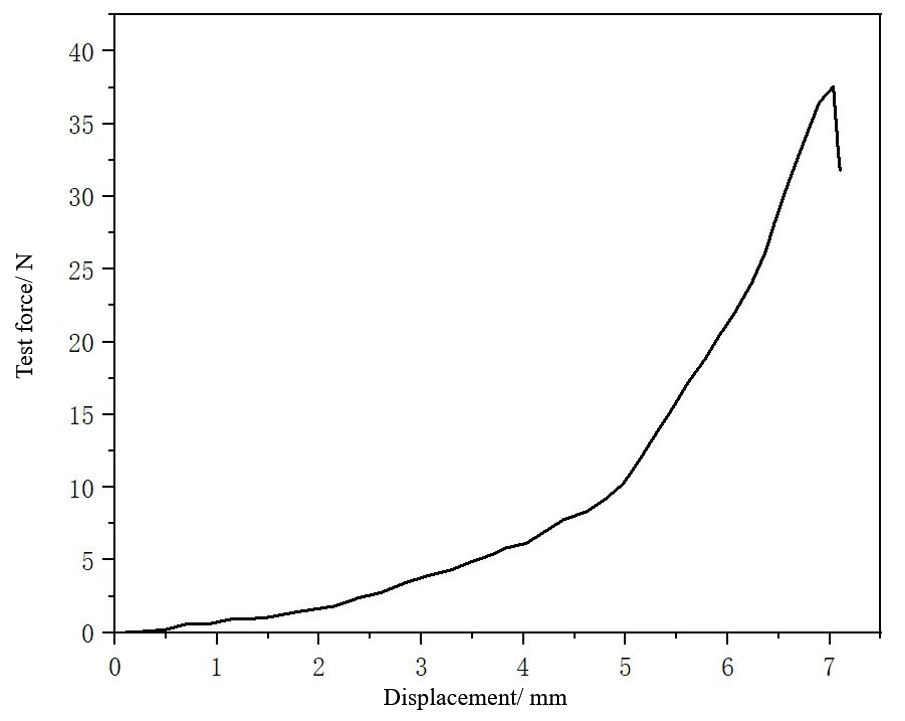


Supplementary Figure 8: Radial Compression Stress-Strain Curve.


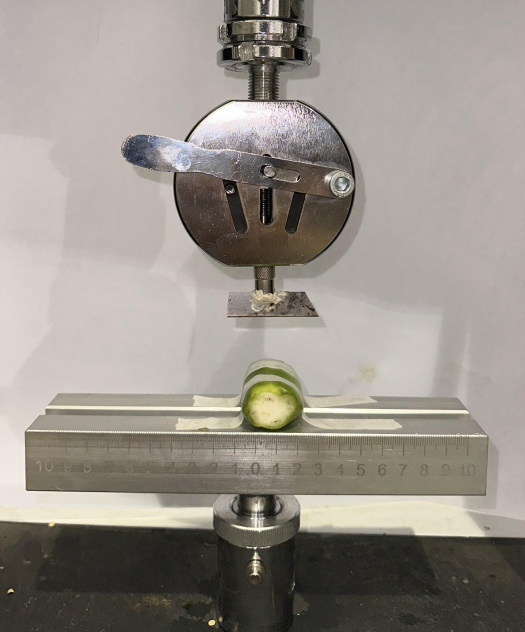


Supplementary Figure 9: Shear Test.


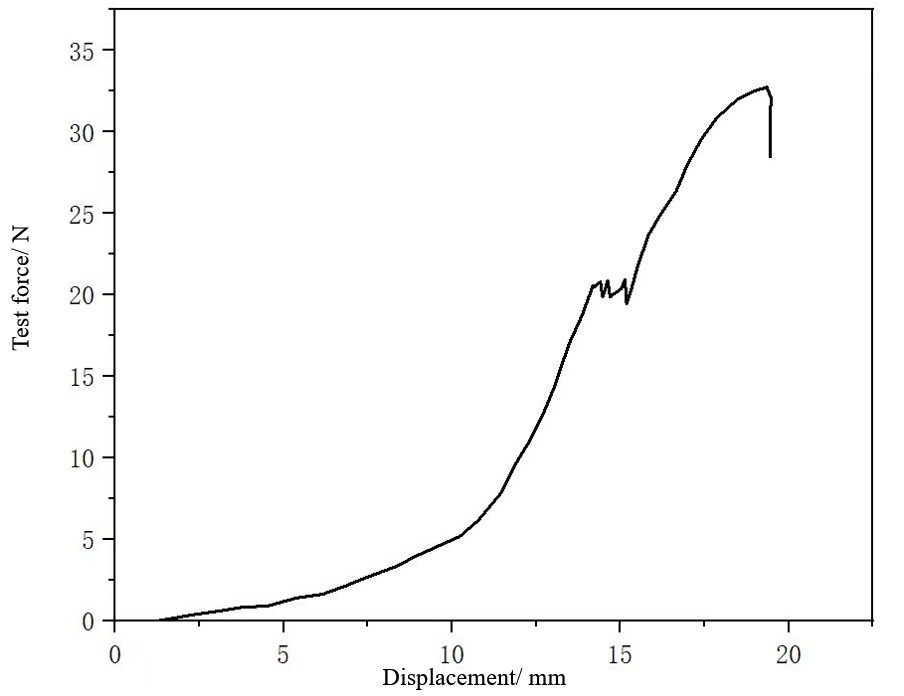


Supplementary Figure 10: Shear Stress-Strain Curve.


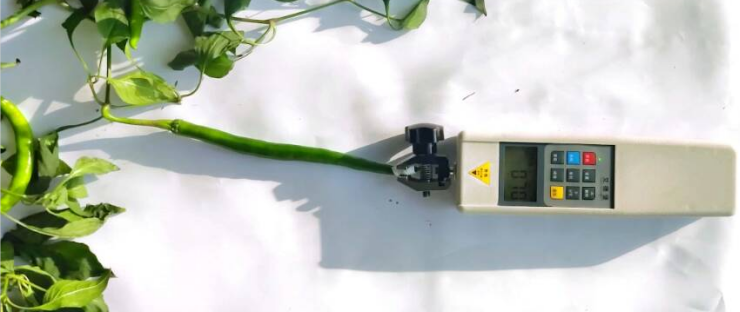


Supplementary Figure 11: Measurement of the connection force between pepper and stem.
